# Quasi Real-Time Apple Defect Segmentation Using Deep Learning

**DOI:** 10.3390/s23187893

**Published:** 2023-09-14

**Authors:** Mirko Agarla, Paolo Napoletano, Raimondo Schettini

**Affiliations:** Dipartimento di Informatica, Sistemistica e Comunicazione, Università Milano-Bicocca, 20126 Milano, Italy; m.agarla@campus.unimib.it (M.A.); paolo.napoletano@unimib.it (P.N.)

**Keywords:** apple defect segmentation, multispectral imaging, real-time deep learning, visual inspection

## Abstract

Defect segmentation of apples is an important task in the agriculture industry for quality control and food safety. In this paper, we propose a deep learning approach for the automated segmentation of apple defects using convolutional neural networks (CNNs) based on a U-shaped architecture with skip-connections only within the noise reduction block. An ad-hoc data synthesis technique has been designed to increase the number of samples and at the same time to reduce neural network overfitting. We evaluate our model on a dataset of multi-spectral apple images with pixel-wise annotations for several types of defects. In this paper, we show that our proposal outperforms in terms of segmentation accuracy general-purpose deep learning architectures commonly used for segmentation tasks. From the application point of view, we improve the previous methods for apple defect segmentation. A measure of the computational cost shows that our proposal can be employed in real-time (about 100 frame-per-second on GPU) and in quasi-real-time (about 7/8 frame-per-second on CPU) visual-based apple inspection. To further improve the applicability of the method, we investigate the potential of using only RGB images instead of multi-spectral images as input images. The results prove that the accuracy in this case is almost comparable with the multi-spectral case.

## 1. Introduction

The food industry is concerned with the processing, production, handling, storage, preservation, control, packaging, and distribution of food products made from raw foods [1]. The early detection of defects in raw food would make food production and selection processes more efficient. Kumar Pothula et al. [2] propose a new singulating and rotating mechanism for in-field grading and sorting, and Nturambirwe et al. [3] and Firouz et al. [4] review the automatic non-destructive techniques for horticultural quality assessment. The detection of defects in this case would allow food prices to be categorized according to product quality, thus better matching consumer expectations with respect to a specific product category [5].

Detecting defects during production, handling, storage, preservation, etc. is crucial for prompting appropriate categorization of the products, especially in the food industry. To this aim, it is essential to detect defects within a timeframe that is shorter or at least comparable to the product processing time itself, thus ensuring that timely feedback or alerts are provided.

Defect detection can be carried out through various methods. First, human operators can perform manual defect detection to actively monitor the manufacturing process. Alternatively, computer-based systems equipped with imaging devices or sensors can automatically monitor the production process. Lastly, there is the option of semi-automated detection, where humans interact with computer-based monitoring systems [6] to analyze critical cases.

In this paper, we focus on the quality control of raw food, which is usually based on shapes, colors, and textures [7], and in particular we address the problem of apple quality control. Defect detection of apples is a relevant problem since the apple is the second most consumed fruit in the world, following the banana [8]. China provides the largest apple production in the world; it produced almost 40 million metric tons with a cultivation area of 2.1 million hectares in 2013. Other leading countries include Turkey, the United States, Poland, and India (see Table 1). The majority of apples produced in the world are destined for fresh consumption, so a defect detection method is crucial to improve the market value of these fruits. Serious or slight defects could be both naturally or non-naturally introduced in the apple life cycle. Natural defects are produced during the fruit’s growth on the tree, for example, frost damage, rot, hail damage, flesh damage, and scald. Non-natural defects are introduced from the harvest operation onwards, for example, spoilage or mechanical damage, transportation spillage, degradation, grading, sorting, washing, and distribution in the stores. Typical non-natural defects are bruises, russets, scar tissue, limb rubs, and flesh damage.

In this paper, we focus on apple defect detection using automatic visual inspection methodologies based on computer vision and deep learning. The majority of the state-of-the-art methods consider apple defect detection as a classification problem, where each apple sample is classified as a defective/non-defective class with any information about the defect localization and size [9]. However, the European Commission in 2004 defined the marketing standard for apples, asserting that apples are classified in three classes on the basis of the grade of defects in terms of size and visual characteristics [10]. In order to be compliant with European standards, automatic methods for apple defect detection should also output the segmentation of the defective regions. Defect segmentation makes it possible that some apples could be sold at a lower price if the defect covers a small portion of the surface.

Huang et al. [11] found that the use of hyper and multi-spectral cameras instead of RGB cameras may bring an improvement in defect detection since some classes of defects are more visible in specific spectral bands. Multi-spectral imaging for raw foods can be performed using two common measurement modes, namely reflectance and transmittance. The difference between them is related to the lighting and detector configurations. The reflectance mode can obtain information concerning the sample surface in terms of color, size, shape, and surface defects, without any contact with it [12]. The transmittance mode measures the amount of light that passes through the sample [13], thus providing information regarding the internal part of the sample. Concerning apples, the reflectance mode is the most common choice because it is non-destructive, fast, simple, low-cost, and environmentally friendly. In this field, Rahi et al. [14] review the spectroscopy and spectral imaging techniques for non-destructive food microbial assessment, Gui et al. [15] propose a CNN-SVM model for an SMV classification method trained on hyperspectral images with 256 bands in the range 383.70∼1032.70 nm, while Liu et al.’s [16] system discriminates and eliminates damaged soybean seeds based on the acquired RGB images.

Notwithstanding the importance of visual-based automatic detection of apple defects, the databases that are used for developing these types of systems are usually not available for research. In this work, we employ the only public available multi-spectral database of apples (reflectance mode: RGB + near infrared-NIR). The database has precise segmentation and categorization of defects [17]. It consists of 280 and 256 images of healthy and defective *Jonagold* apples, respectively. The multi-spectral (RGB + NIR) images are acquired in a custom setup that includes a four band multi-spectral image acquisition device and a specific set of lighting sources.

Before the emergence of convolutional neural networks (CNNs), traditional computer vision techniques were commonly used for defect segmentation tasks. These methods often relied on handcrafted features and rule-based algorithms to detect and segment defects on apple surfaces. Here are a few techniques that were commonly used prior to the widespread adoption of CNNs: Mizushima and Lu [18] propose an image segmentation algorithm for apple sorting and grading based on support vector machine and Otsu’s method; Unay et al. [19] propose several thresholding and classification-based techniques for defect segmentation on ‘Jonagold’ apples; Kleynen et al. [17] implement filters of specific spectral bands for a multi-spectral image acquisition device for defect detection on apples.

Convolutional neural networks (CNNs) have emerged as a powerful approach for defect segmentation tasks, including the specific task of apple defect segmentation. CNNs are deep learning models that excel at analyzing visual data and have the ability to automatically learn and extract relevant features from images [20].

By utilizing CNNs for apple defect segmentation, researchers have achieved significant improvements in accuracy and efficiency compared to traditional computer vision techniques. CNNs can handle complex and varied defect patterns, adapt to different lighting conditions, and learn robust representations of the defects, making them a state-of-the-art approach in this field [21].

To train these CNN models, large datasets of annotated apple images are required. These datasets contain images of apples with different types and severities of defects, along with corresponding manual annotations that indicate the locations and boundaries of the defects. The process of creating these annotated datasets involves expert human annotators who carefully label the defects in the images [17].

The main limitation of deep neural networks for defect detection is related to the amount of diversity data in the training process. Acharya et al. [22] generate synthetic data to overcome imbalance problems. Recent works increase the number of training images by applying different traditional augmentation techniques like salt and pepper noise, Gaussian noise, flips, rotation, brightness, and darkness operation [23]. But these augmentation algorithms are not enough to increase the diversity of defects in the database and they can change the naturalness of defects.

Moreover, relevant problems of CNNs are related to the inability to run on low computational devices and handle high resolution images in real-time. The most straightforward solution to reduce the computational complexity is to resize the image, but this operation introduces some artifacts and can alter the defects [24].

This paper proposes an advanced deep learning methodology for the automatic segmentation of apple defects. Our approach is based on a U-shaped convolutional neural networks (CNN) architecture [25]. To address the challenge of limited training data and thus reduce the effect of the training overfitting, we propose here a novel data synthesis technique. This technique aims to increase the number of available samples for training. To assess the effectiveness of our approach, we conduct evaluations using the multi-spectral apple dataset proposed by [17].

Our proposed methodology exhibits superior performance in terms of segmentation accuracy compared to commonly used deep learning architectures designed for general segmentation tasks. Our approach significantly improves existing methods for apple defect segmentation, leading to substantial enhancements. Additionally, we evaluate the computational cost of our proposal, demonstrating its suitability for real-time applications. Utilizing a GPU, our method achieves an impressive frame-per-second rate of approximately 100, while with a CPU, it achieves a quasi-real-time performance of about 7/8 frames-per-second in visual inspection processes. To enhance the versatility of our method, we investigate the feasibility of using RGB images exclusively as input data instead of multi-spectral images. Encouragingly, the results show that the accuracy achieved in this scenario is nearly comparable to the multi-spectral approach. The experiments can be reproduced using the code made available at the following address: https://github.com/cimice15/Quasi_real-time_apple_defect_segmentation (accessed on 8 September 2023).

The paper is organized as follows: Section 2 presents related works, Section 3 presents the database used in our experiments and the method we propose. Section 4 presents evaluation metrics and experimental setups. Finally Section 5 discusses results of the proposed method in comparison with state-of-the-art methods.

## 2. Related Works

We approach the apple quality assessment as a defect segmentation task because binary classification restricts the possible performance evaluation and the value on the market of the algorithms. So in this section we analyze the methods specifically designed for the apple defect segmentation and the methods that perform apple classification exploiting defect segmentation. The result of this analysis is reported in Table 2.

Unay et al. [19] propose an approach that compares several thresholding and classification-based techniques for apple defect segmentation. The method extracts global and local apple features using different neighbourhoods size and shape. Subsequently, the defect detection is conducted using thresholding or classification. Thresholding applies global or local threshold, while classification uses supervised and unsupervised methods. The results shows that the multi layer perceptron (MLP) is the most promising architecture to be used for the segmentation of surface defects.

Xiaobo et al. [31] propose a multi-threshold method to segment the apple image from black background. Then, the Yang et al. [32] algorithm identifies patch-like defects including calyxes and stem-ends. When two or more *ROI*s are identified, the apple is classified into the rejection class. In this case, the segmentation of the defect is a previous stage of the apple classification.

Zhang et al. [26] propose an approach for identifying defects in apples using a combination of the fuzzy C-means algorithm and the nonlinear programming genetic algorithm (FCM-NPGA), along with multivariate image analysis. Initially, the image was subjected to denoising and enhancement through fractional differentiation. This process eliminated noise and edge points while retaining essential texture details. Subsequently, the FCM-NPGA algorithm was employed to segment potentially defective regions within the apple. Ultimately, a strategy founded on multivariate image analysis was employed to identify flaws within the mapped regions indicative of potential defects in the apples.

Bhargava et al. [29] applied a threshold to segment apple instance from background, then the segmentation of a defective area is performed using fuzzy c-means. The method is designed for apple classification, so the apple feature extraction is performed using various combinations of statistical textural, geometrical, Gabor wavelet, and discrete cosine transform. Finally, for classification, three different classifiers, namely KNN, SRC (sparse representation classifier) and SVM (support vector machine) have been applied.

Huang et al. [11] developed a multi-spectral imaging system to select the most appropriate wavelengths for apple defect classification using principal component analysis (PCA). Although the method is developed for the classification of normal or bruised apples, the analysis highlights that three effective wavelengths are feasible for bruise segmentation on apples.

Lu et al. [28] developed a multi-spectral structured illumination reflectance imaging (SIRI) system to acquire near-infrared images of apples with various types of surface and subsurface defects. Direct component (DC) and amplitude component (AC) images are extracted and enhanced using bi-dimensional empirical mode decomposition (BEMD). Defect detection algorithms are developed using random forest (RF), SVM, and CNN.

The most recent method proposed by Fan et al. [30] combines NIR images provided by three consecutive rubber roller stations. Then, the defect detection and classification are performed using a pruned YOLO V4 network.

Since our method is based on a U-shaped CNN architecture, we compare our method with other U-shaped architectures as well as with other relevant CNN architecture specially designed for image segmentation. In particular, we compare with a traditional U-Net [33] and a variant, namely U-Net++ [34], the Pyramid Scene Parsing Network (PSPNet) [35], DeepLabv3 [36] and PAN (Pyramid Attention Network) [37]. The U-net network consists of a contracting path to capture context and a symmetric expanding path that enables precise localization. Its variant U-Net++ is aimed at reducing the semantic gap between the feature maps of the encoder and decoder sub-networks through a series of nested and dense skip pathways. PSPNet embeds difficult scenery context features in a fully convolutional network-based pixel prediction framework. DeepLabv3 network employs atrous convolution in cascade or in parallel to capture multi-scale context by adopting multiple rates. Finally, the PAN network combines attention mechanism and spatial pyramid to extract precise dense features for pixel labeling instead of complicated dilated convolution and artificially designed decoder networks.

## 3. Materials and Methods

### 3.1. Database

The apple image database [17] consists of 280 and 256 images of healthy and defective *Jonagold* apples, respectively, with a resolution of 430×560 pixels. The multi-spectral (RGB + NIR) images are acquired in a custom setup that includes a four band multi-spectral image acquisition device and a specific set of lighting sources. The acquisition device is a MultiSpec AgroImager^TM^ (Optical Insights LCC, Suwanee, GA, USA) with four interference band-pass filters centred at 450, 500, 750 and 800 nm with a bandwidth of 80, 40, 80 and 50 nm, respectively. Each band is 8-bit encoded. The acquisition lighting setup is composed by two Philips TL-D 18 W/18 fluorescent tubes emitting in the spectral band of the blue colour and ten 30 W incandescent spots emitting in the visible and near-infrared spectra. Lighting sources are placed to avoid specular reflections.

**Figure 1:**
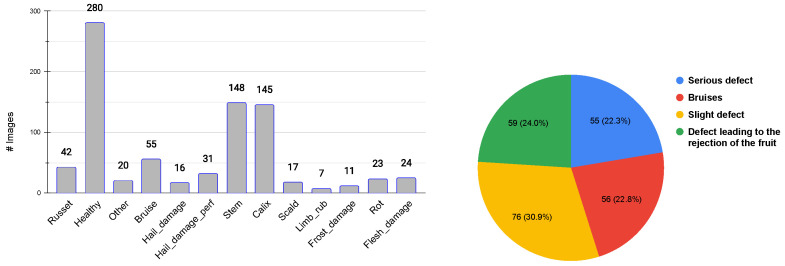
Distribution of defect classes (**left**) and distribution of defects based on their severity and size (**right**).

**Figure 2:**
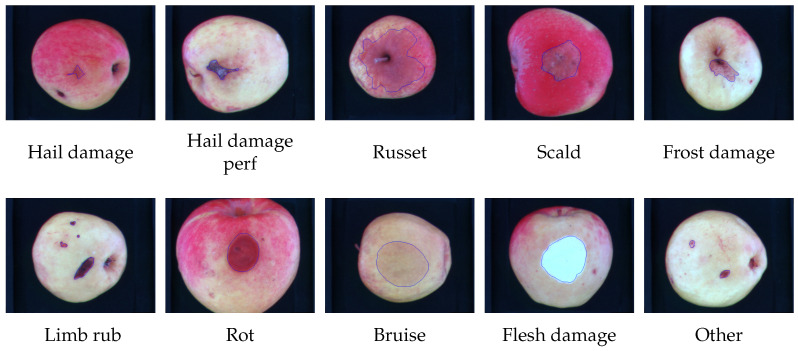
Examples of apple defects present in the database. The dataset is composed of 9 classes of defect plus an *other* class. For each image the limited blue area highlights the defect area.

The apples in the database contain a large number of defect variants both in terms of appearance and size: *russets*, *scar tissue*, *frost damage*, *scald*, *hail damage* (with and without skin perforation), *limb rubs*, *visible flesh damage*, *recent bruises* (between 1 h and 2 h old), *rot* and other (defects). Individual defects are further characterized, according to their severity and size, as follows: slight defects (e.g., small russet), more serious (e.g., scar tissue), defects leading to the rejection of the fruit (e.g., rot) and recent bruises. The defects distribution is depicted in Figure 1. In the original database, all the defects have been manually outlined, making it possible to faithfully verify the accuracy of the proposed method that is designed to automatically identify the boundaries of defective areas whatever the type of defect is. An example of these defects is depicted in Figure 2.

**Figure 3:**
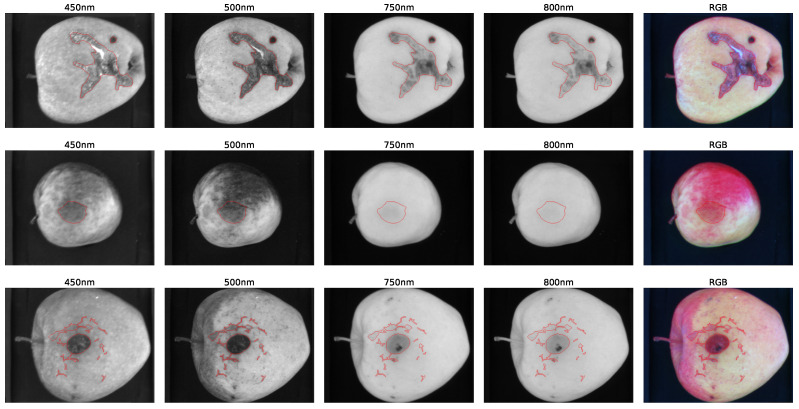
Apple images with the corresponding defect segmentation. Each column presents the apple images at a given band (450, 500, 750 and 800 nm). The last column presents the corresponding RGB image. For each image the defective regions are highlighted in red. Each row displays apples with different defect types.

Figure 3 shows the spectral bands of some defective apples. It can be noticed that some defects are difficult to see in some spectral bands. Since multispectral cameras are usually more expensive than traditional RGB cameras, one of the research questions to be addressed in concerns the actual need of the infrared channel for defect segmentation.

### 3.2. Data Synthesis

To further increase the number of apple samples used during the training process, and so to reduce overfitting of the neural networks, we synthesize new defective apple images. The data synthesis process is summarized as follows:

1. We randomly pick a defective apple image;
2. We apply two image transformations to the apple defect: (1) a random rotation of an angle selected within the range [0,360]deg; (2) a random warping using a deformation grid [33];
3. We randomly pick a healthy apple image;
4. We define the apple Region Of Interest (*ROI*) by removing the background from the healthy apple image [19];
5. We place the defect taken from the defective apple image selected at step 1 onto the *ROI* of the healthy apple image.

To synthesize apples with multiple defects, we randomly place from one to three defects onto a given healthy apple image.

To further analyze the effect of our data synthesis procedure, we have investigated different data synthesis setups that are listed in Table 3. The first setup considers no data synthesis at all; the second one considers only the generation of one synthetic defect without any transformation; the third one considers one synthetic defect with random defect rotation; the fourth one considers one synthetic defect with random defect rotation and warping; the last two setups consider one to three synthetic defects along with defect rotation and both defect rotation and warping, respectively.

**Figure 4:**
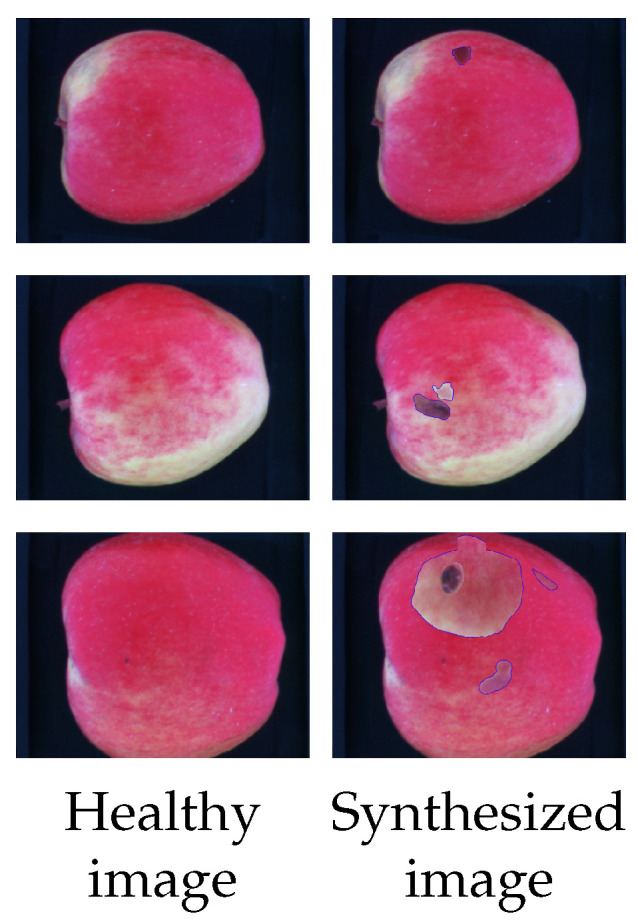
Examples from our data synthesis procedure. Each row represents a different healthy image that is synthesized with 1, 2, and 3 defects respectively coming from non-healthy apples. For each image the the limited blue area highlights the defect area.

The proposed data synthesis procedure is executed during the training phase with a probability P. A graphical example of the proposed data synthesis procedure (setup number 5, cfr. Table 3) is depicted in Figure 4. Each row corresponds to a different defect type, while the first and second columns represent healthy apple without and with synthesized defects, respectively.

### 3.3. Proposed Model

Our approach is based on a U-shaped CNN architecture that has been successfully employed in the literature for visual inspection tasks [38]. This architecture is a good trade-off between accuracy and computational cost [33]. We show later that our proposal can be employed in real-time (using a GPU) or quasi-real-time (using a CPU) visual inspection tasks. Moreover, the network designed here allows the handling of full size images without resizing and cropping that can alter defect details [39].

**Figure 5:**
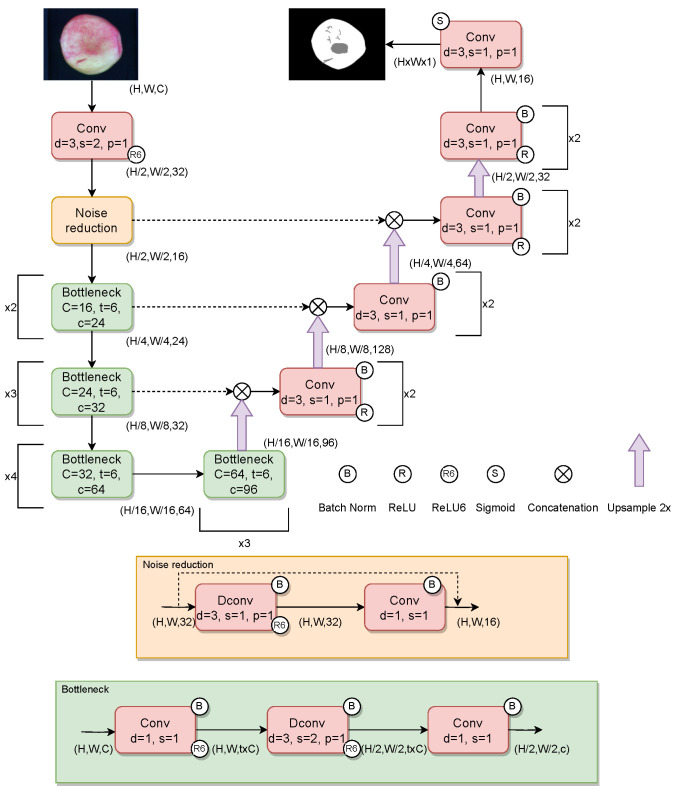
Our proposed model architecture. Regarding the convolution operation, *d*, *s* and *p* refers to the square kernel size, stride and padding, respectively. The *C*, *t* and *c* parameters of bottlenecks indicate the input channels, the expansion ration of channels in the middle depth-wise convolution and the output channels, respectively.

The proposed U-shaped architecture is depicted in Figure 5. The first two blocks of the network encoder are designed for feature mapping and noise reduction. The feature mapping is performed using a convolution layer that increases the number of features and halves the image size so to reduce the computational cost. Then, the noise reduction module is based on an inverted residual structure that is performed using a depth-wise convolution and a point-wise convolution that halves the number of channels to remove irrelevant features. Subsequently, the next group of bottleneck layers perform a channel feature selection, where each bottleneck block is composed of three operations: firstly the number of features is expanded by a factor of *t* using a point-wise convolution; secondly, a depth-wise convolution with 3×3 kernel of stride 2 halves the image size; finally the number of features is reduced using a point-wise convolution. The last bottleneck layer performs a feature mapping for the decoding phase. The number of bottleneck blocks increase as the spatial dimension decreases in order to catch large details like bruises and fine grain details like russet or scald, respectively. During the decoding phase, the features of the lower level are up-sampled and concatenated with the features of the level above. Then a 3×3 convolution layer with a stride 1 and padding 1 is applied in order to efficiently combine the information of the two different levels without changing the number of features. Batch normalization and a *ReLU6* non linearity function follow each layer to encourages the model to learn sparse features earlier.

To train our proposed architecture, we employ the Focal Tversky Loss (FTL) [40] that permits to improve the *precision* and *recall* balance in semantic segmentation when dealing with highly imbalanced dataset. The FTL gives better results than other binary classification loss functions, such as the Binary Cross-Entropy, Weighted Cross-Entropy and Dice Loss [41]. The FTL is defined as follows:

(1) $$\begin{array}{cc} TI_{c}= & \frac{\sum_{i=1}^{N}p_{ic}g_{ic}+\epsilon }{\sum_{i=1}^{N}p_{ic}g_{ic}+\alpha \sum_{i=1}^{N}p_{i\bar{c}}g_{ic}+\left(1-\alpha \right)\sum_{i=1}^{N}p_{ic}g_{i\bar{c}}+\epsilon }\\ FTL_{c}= & \sum_{c}{\left(1-TI_{c}\right)}^{1/\gamma }, \end{array}$$

where $p_{ic}$ is the probability that pixel *i* is predicted as defective *c* and $p_{i\bar{c}}$ is the probability that a pixel *i* is healthy $\bar{c}$. $g_{ic}$ and $g_{i\bar{c}}$ is the pixel *i* ground-truth of the defective and healthy class respectively. *N* is the total number of pixels in the image. The ϵ provides numerical stability to prevent division by zero. The α parameter ranges in {0,1} and it is used to train a more *recall* or *precision* oriented model. The γ coefficient allows the model to focus more on less accurate predictions that have been misclassified, such as those with small ROIs.

#### Comparison with the State-of-the-Art

To study the impact of different architectures in the detection of apple defects, we compare our method with pixel-wise and CNN state-of-the-art models. The most relevant pixel-wise approach in the state-of-the-art that experiments with the apple dataset under investigation is proposed by Unay et al. [19]. Unlike convolutional neural networks, one of the problems of pixel-wise methods is that they do not consider spatial correlation among pixels in the detection of the defects which is indeed a relevant information in semantic segmentation.

We compare our proposed architecture with several variants of U-shaped CNNs commonly used in binary and semantic segmentation tasks. The first variant is a traditional U-Net architecture that consists of three downsampling layers with 3×3 convolution followed by an average-pooling layer of size 2×2. In this architecture and the following models the output image is processed with a sigmoid activation aimed to get defect probabilities. In order to reduce the semantic gap between the feature maps of the U-Net encoder and decoder sub-networks, we experiment a U-Net++ model [34]. This architecture is based on nested and dense skip connections between intermediate blocks, making the model more accurate in image segmentation. The size of convolution filters and up-sampling layers of U-Net++ are the same as those of the traditional U-Net.

We also include in our comparison some relevant CNN architectures for semantic image segmentation: Pyramid Scene Parsing Network (PSPNet) [35], DeepLabv3 [36], and PAN (Pyramid Attention Network) [37].

## 4. Experimental Setup

### 4.1. Evaluation Metrics

For the evaluation of our experiments we adopt traditional metrics, such as *recall*, *precision*, *f*-score [42] and a special metric, namely Class-Specific Recognition Error (*CSRE*), that takes into consideration the fact that defects may be very small [19] with respect to the size of the apple.

Given an apple image, the *CSRE* is defined as follows:

(2) $$CSRE=\frac{\frac{FN}{TP+FN}+\frac{FP}{TN+FP}}{2},$$

where *TP* is the number of defective pixels correctly detected, *FP* is the number of healthy pixels incorrectly detected as defect, *TN* is the number of healthy pixels correctly detected, and *FN* is the number of defective pixels incorrectly detected as healthy.

The *f*-score is defined as follows:

(3) $$f-\mathrm{score}=\frac{precision\cdot recall}{precision+recall}=\frac{2TP}{2TP+FP+FN}.$$

The *precision* and *recall* scores for each method are as follows:

(4) $$precision=\frac{TP}{TP+FP}\;recall=\frac{TP}{TP+FN}.$$

### 4.2. Experiment Design

We conducted preliminary experiments with our solution adopting a 3-fold Cross Validation (CV). Results with CV were quite stable across folds, suggesting the affordability of using only one fold as test set so to saving computational resources. In fact, our experiments included the training of seven different neural network models using two different color spaces with and without data augmentation, resulting in 28 training from scratch. Moreover, for the sake of comparison, we also conducted an ablation study of our data synthesis method that required eight, training from scratch the proposed neural network. Finally the total number of training processes is about 36. For all the experiments, we divide the dataset into 2 folds: 67% for training/validation and 33% for testing. Training data are further split into two sets containing actual training samples (50%) and validation samples (17%).

The experimental setup relative to Unay et al. [19] follows the steps and the optimal parameters provided in the original paper. For the training of all the CNN-based methods (including our proposal) we adopt the same experimental setup. We set the max number of epochs to 100 with early stopping of 15 epochs of no improvements on validation set. Adam is used as the optimizer starting from $lr=1\times 10^{-4}$, β(0.9,0.999), $\epsilon =1\times 10^{-8}$ and weight decay is set to 0. We schedule the learning rate with step of 0.7 every 10 epochs. FTL, Equation (1), is used as loss function with γ=0.75 and α=0.5 as base configuration [40].

Our data synthesis procedure is applied with a probability P equal to 80%. In addition, a traditional data augmentation techniques is applied to each image with 50% probability: horizontal and vertical flip, and rotation [0,90deg] each with 50% probability are applied.

## 5. Results and Discussion

In this section, we present and discuss the results achieved with our proposal and the comparison with the state-of-the-art in all the experimental setups. Before going into details, we show Table 4 which summarizes all the experiments and comparisons. The table shows the rank of all the experimented algorithms computed averaging the ranks with respect all the evaluation metrics and across all experimental setups. Overall, our proposal ranked at first position whatever the input data are (RGB or RGB + NIR) and whether or not data augmentation is used, thus demonstrating the superiority of our proposal with respect to the state-of-the-art. The second best method is DeeplabV3 while the third one is PAN. The worst one is the method by Unay et al. [19], which is based on handcrafted features and an MLP.

### 5.1. Hand-Crafted and CNN-Based Methods in the State-of-the-Art

Table 5 shows the results in terms of *CSRE*, *f*-score, *precision*, *recall* of the proposed method, and the state-of-the-art. We report on the use of RGB + NIR and RGB solely so that we can evaluate the potential of using only RGB images instead of multi-spectral ones. Overall, our method outperforms in terms of *CSRE* of about 1% and 16% CNN-based methods and the hand-crafted method, respectively. In terms of *f-score*, our method outperforms about 3% and 34% CNN-based methods and the hand-crafted method, respectively. Among CNNs, the second best approach is the Deeplab V3, which is based on a multi-scale processing that helps to catch fine and coarse defects.

At this stage, it is important to highlight that the proposed data synthesis procedure is adopted only in our CNN architecture, while traditional data augmentation is employed in all CNNs. Later on (cfr. Section 5.5), we show the effects of the proposed data synthesis procedure when applied to CNNs in the state-of-the-art.

Finally, the results show that RGB + NIR and RGB perform in a very similar way thus showing the potential of using only RGB cameras in apple visual inspection instead of multi-spectral cameras.

**Figure 6:**
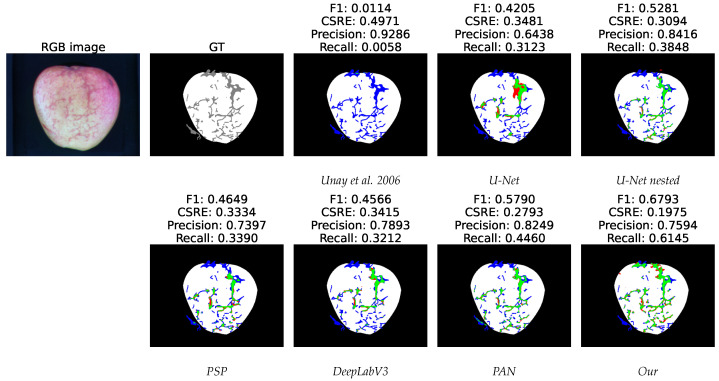
Predicted segmentation of RGB + NIR russet defective apple with the investigated methods. The first two images are RGB image and respective ground-truth segmentation. Regarding the segmentation performed by the models, true positives, false positives, and false negatives are showed as green, red and blue color respectively. For each method we report *f*-score, *CSRE*, *precision* and *recall* of the segmentation with respect to the ground-truth [19].

Figure 6 shows the segmentation output of a russet defective apple with the investigated methods in the RGB + NIR configuration. The first two images are RGB image and the respective ground-truth segmentation. Regarding the segmentation performed by the models, true positives, false positives, and false negatives are showed as green, red and blue color respectively. For each method we report *f*-score, *CSRE*, *precision* and *recall* of the segmentation with respect to the ground-truth. Our method outperforms by large improvement the state-of-the-art models, specially for the smaller defective areas.

### 5.2. Comparison with Hand-Crafted and CNN-Based Methods in the State-of-the-Art in Terms of Macro Class and Sub Class Defects Accuracy (RGB + NIR and RGB)

Table 6 and Table 7 show the comparison between our method and the state-of-the-art in terms of accuracy of the macro classes of defects when RGB + NIR and RGB are employed, respectively. For what concerns RGB + NIR, we outperform the state-of-the-art in terms of *f*-score on all classes apart from the class *bruises* where Deeplab v3 is about 3% better than our solution. In contrast, in the case of RGB, we outperform in terms of both *f*-score and *CSRE* the state-of-the-art on all macro classes of defects.

Table 8 and Table 9 show the comparison between our method and the state-of-the-art in terms of accuracy of the sub classes of defects when RGB + NIR and RGB are employed respectively. For what concerns RGB + NIR, on average we outperform the state-of-the-art in terms of *f*-score, but we perform much worse than Deeplab v3 on the class *other* that includes defects with a very low impact to the visual appearance of the apple and that rarely have effects to the inner part of the apple. However, in the case of RGB the *other* class of defects is detected with a higher *f*-score with respect to the case RGB + NIR and very close the best method (below about 1%). This result also confirms the goodness of the proposed method when only RGB input is processed with respect to the multi-spectral one.

### 5.3. Sensitivity of the Proposed Method to the Parameter α of the TFL

**Figure 7:**
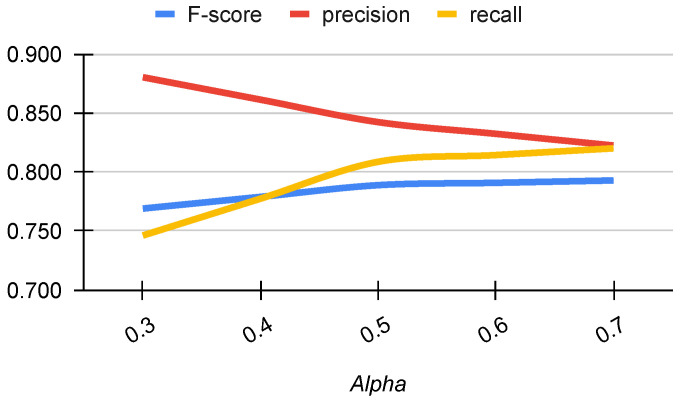
The study of α (Alpha) parameter of FTL.

The Focal Tversky Loss (FTL), Equation (1), performs a combination of False Positives (*FP*) and False Negatives (*FN*). The α parameter represents a trade-off between *FP* and *FN*, thus the tuning of this parameter allows to obtain a more precision or recall oriented model, Figure 7. The model becomes more recall oriented as we increase the value of α, while the model becomes more precision oriented as we decrease the value of α. The behavior of the *f*-score highlights that the model performs in a good way starting from a value of α of about 0.5 that corresponds to our choice.

### 5.4. Data Synthesis Ablation Study (RGB + NIR and RGB)

Table 10 shows the comparison between different variants of the proposed data synthesis algorithm applied to our model when the input is RGB. The first row in the table corresponds to no data synthesis at all. The second variant adds one synthesized defect to the healthy apple without any rotation and warping of the defect. The third and forth variants consider also the rotation and warping of the defect. Finally, the fifth and the sixth consider multiple synthesized defects (from 1 to 3) along with rotation and warping.

Results show that defect rotation improves all metrics, while the defect warping operation does not improve the performance. This is due to the warping operation that adds pixel artifacts in order to perform the defect deformation. Overall, considering multiple synthesized defects instead of a single synthesized defect improves slightly improves the *f*-score and *precision*. The best algorithm, in terms of evaluation metrics, is the number 5, where the synthesized apple has 1 to 3 defects that are randomly rotated. To the sake of comparison, we show the difference between the variant number 1 and number 5 when the input is RGB + NIR. Also in this case, it is clear the improvement we achieve with our data synthesis methodology.

### 5.5. Comparison with CNN-Based Methods in the State-of-the-Art That Use Data Synthesis (RGB + NIR and RGB)

To reduce the effect of the training overfitting, we apply the proposed data synthesis technique also to the state-of-the-art methods. This technique aims to generate different images, thus increasing the number of available samples for training. Table 11 shows the results in terms of *CSRE*, *f*-score, *precision*, *recall* of the proposed method and the CNN-based state-of-the-art where the proposed data synthesis is applied. For all methods, the variant number 5, that is the one with multiple synthesized defects (from 1 to 3) along with rotation, is applied. We report on the use of RGB + NIR and RGB solely so that we can evaluate the potential of using only RGB images instead of multi-spectral ones. The use of data synthesis improves the mean performance of *CSRE* by RGB and RGB + NIR configurations. Our method outperforms the state-of-the-art in terms of *CSRE*, *f*-score and *recall*, but we perform worse than PAN on *precision* in the case of RGB + NIR. It is interesting to note that the PAN approach with data synthesis becomes the second best along with Deeplab V3.

### 5.6. Analysis of the Computational Cost

To assess the applicability of the proposed method in real-world scenarios, we measure and compare the computational cost of the proposed method and state-of-the-art. Experiments are performed on a workstation equipped with an Intel Core i7-7700 CPU@3.60 GHz, 16 GB DDR4 RAM 2400 MHz, and NVIDIA Titan Xp with 3840 CUDA cores. The operating system is Ubuntu 22.04. All methods are implemented using Python and the Deep Learning framework Pytorch.

**Figure 8:**
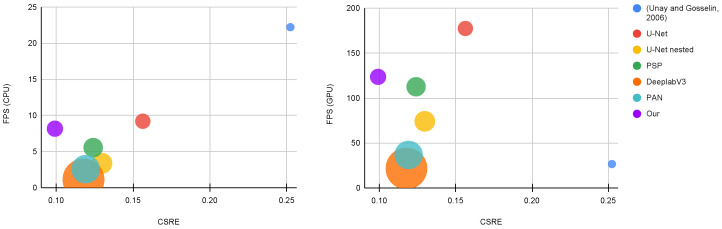
Methods FPS, *CSRE*, and FLOPS(G) comparison of RGB + NIR configuration. The first and the second plots are related to the CPU and GPU configurations, respectively. Top-left configuration is better.

The results are presented in Table 12 and depicted in Figure 8. The table shows Class-Specific Recognition Error (*CSRE*) [19], Frame Per Seconds (FPS) when the method is run on CPU and GPU, and the number of Floating-Point Operations (FLOPS) measured in billions (G). For the FLOPS and *CSRE* metrics, lower is better, while for the FPS higher is better. This comparison is done considering RGB + NIR as input. The color grading from red to green stands for worse and better behavior, respectively. Results show that the hand-crafted method is the one with the lowest computational cost but with the worst *CSRE*. Amongst the CNN-based approaches, our proposal has the best trade-off between *CSRE* and computational cost. It permits to achieve up to 124 FPS with about 7 GFLOPS and a *CSRE* lower than 0.1. Figure 8 graphically summarizes this comparison in terms of *CSRE* vs FPS measured on CPU and GPU. The size of the bubbles refers to the computational cost. The larger is the bubble the larger is the computational cost. This picture clearly shows that our method is the one with the best trade-off between performance and computational cost.

## 6. Final Remarks and Conclusions

In this paper, we proposed a novel deep learning approach for the automated segmentation of apple defects using a convolutional neural network (CNN) based on a U-shaped architecture with skip-connections only within the noise reduction block. To increase the number of samples and at the same time to reduce neural network overfitting, we designed an ad-hoc data synthesis technique to generate new images of apples having a large variety of defects. We compared the proposed approach, both with and without data synthesis, with different CNN-based methods, namely Pyramid Scene Parsing Network (PSPNet) [35], DeepLabv3 [36] and PAN (Pyramid Attention Network) [37], and a hand-crafted method proposed by Unay et al. [19]. To further improve the applicability of the method, we also investigated the potential of using only RGB images instead of multi-spectral (RGB + NIR) ones.

The results show that all the experimented CNN-based approaches outperform, in terms of *f*-score, of about 35% the hand-crafted algorithm proposed by Unay et al. [19] in both RGB and RGB + NIR configurations. This behavior was expected since the method by Unay et al. [19] is pixel-wise so it does not consider spatial correlations that are typical in small defective regions. On the contrary, CNN-based approaches process the input image using different receptive fields that permit taking into account spatial correlations among defective pixels.

Our method outperforms, in terms of *f*-score, the other CNN-based approaches by an average of about 6% (the worst of about 15% and the second best of about 2%) with the RGB + NIR configuration. The gap between our method and other CNN-based approaches is much more evident in the case of RGB, which is on average about 9%. Our architecture requires fewer computations with respect to the other CNN-based approaches we experimented with, since it includes a few bottlenecks and uses skip-connections only within the noise reduction block. Since the dataset is of a small amount of defective apples, neural models with a lower capacity are more likely to work better.

To demonstrate that the goodness of our approach does not rely only on the use of the proposed data synthesis procedure, we also experimented with it in combination with the other CNN-based approaches. Results show that, on average, data synthesis permits an improvement of the performance of all CNN-based approaches we experimented with. However, the best of the these CNN-based approaches achieves a performance that is still lower than that of our proposal, so demonstrating the goodness of our architecture. In fact, our method achieves an *f*-score about 4% higher than the state-of-the-art (the worst of about 9% and the second best of about 1%) with RGB + NIR configuration. In the case of RGB the average increment is about 3%.

Experimental results confirm that using learning-based methods instead of hand-crafted ones enable the use of RGB solely instead of RGB + NIR thus enabling the use of conventional cameras in a visual-based apple inspection pipeline. Conventional RGB cameras are at a lower cost with respect to multi-spectral ones and, more importantly, they permit fastening of the processing since the amount of information to be processed is lower.

Finally, the approach we present has the potential to significantly impact automatic visual inspection applications that face the following critical challenges: constrained computational resources and a scarcity of annotated data. To satisfy these real-world constraints, hand-crafted computer vision techniques for feature extraction are applied in conjunction with traditional machine-learning classifiers such as support vector machines. We are all aware that convolutional neural networks can obtain better results. However, these methodologies require large annotated datasets for the training process and significant computational resources at both the training and operating stages. In this regard, our proposed method obtains results that allow us to overcome the aspects mentioned above. Firstly, the superiority of deep neural networks over hand-crafted methodologies demonstrates the capability of our data synthesis technique as a tool to mitigate the lack of training data for these networks. Secondly, our neural network outperforms the state-of-the-art alternatives, in terms of both the accuracy and efficient utilization of computational resources, demonstrating that a well-engineered neural network architecture can give rise to tailored solutions capable of satisfying the computational constraints commonly encountered in real-time applications.

In this work we focused on segmentation, as precise as possible, of defects. Defect classification is at this point much more easier both with neural and hand crafted methods. Our segmentation and data augmentation methods could also be used on other types of fruit.

## Figures and Tables

**Table 1:** Apple production (in 1000 metric tons) by world countries. FAOSTAT, 2023. FAO Statistics Division. https://www.fao.org/ (accessed on 8 September 2023).

Country	2021	2020	2019
China	45,983	44,066	42,425
Turkey	4493	4300	3618
United States	4467	4665	5028
Poland	4067	3555	3080
India	2276	2814	2316
World	93,144	90,490	87,509

**Table 2:** The main state-of-the-art methods for defect segmentation. For each method, the database, the color space, the spectral bands, the methodology, the output and finally the performance are specified.

Author	Database	Color Space	Spectral Bands (nm)	Approach	Output	Performance
Unay et al. [19]	526 Jonagold	Multi-spectral	450, 500, 750, 800	Pixel features + MLP	Binary mask	*CSRE*: 17%
Zhang et al. [26]	2000 Apples	RGB	N/A	FCM-NPGA algorithm	Label	Accuracy: 98%
Huang et al. [11]	250 Fuji	Hyper-spectral	325–1100	PCA + Threshold	Label	Accuracy: 95.3%
Moallem et al. [27]	120 Apples	YCbCr, RGB	N/A	Threshold + SVM, MLP, KNN	Label	Accuracy: 89.2%
Lu et al. [28]	318 Delicious 250 Golden delicious	Multi-spectral SIRI	730	BEMD + RF, SVM, CNN	Label	Accuracy: 98%
Bhargava et al. [29]	526 Jonagold	Multi-spectral	450, 500, 750, 800	Threshold + KNN, SRC, SVM	Label	Accuracy: 95.21%
Fan et al. [30]	610 Apples	RGB + NIR	850	YOLOV4	Label + BBox	Accuracy: 93.9%

**Table 3:** Ablation study of different proposed data synthesis setups.

Algorithm	Synthesized Defects	Rotation	Warp
1	-	-	-
2	1	-	-
3	1	✓	-
4	1	✓	✓
5	1 to 3	✓	-
6	1 to 3	✓	✓

**Table 4:** Methods ranking across experimental setups.

Methods	Aug TS	Unay et al. [19]	U-Net	U-Net Nested	PSP	DeeplabV3	PAN	Our
RGB	no	7	6	4	5	3	2	1
RGB + NIR	no	7	6	5	4	2	3	1
RGB	yes	7	6	5	4	2	3	1
RGB + NIR	yes	6	5	3	4	2	2	1

**Table 5:** Comparison with hand-crafted and CNN-based methods in the state-of-the-art. The methods are evaluated in RGB + NIR and RGB configuration. For each method we report *CSRE*, *f*-score, *precision* and *recall*. Down-arrow and up-arrow indicates lower is better and higher is better for each metric, respectively. In each column, the best and second-best values are marked in **boldface** and underlined, respectively.

Image Type	Method Name	*CSRE* ↓	*f*-Score ↑	*Precision* ↑	*Recall* ↑
RGB + NIR	Unay et al. [19]	0.2523	0.4398	0.5238	0.5166
	U-Net	0.1563	0.6392	0.689	0.7023
	U-Net nested	0.1297	0.7292	0.7947	0.7508
	PSP	0.1240	0.7445	0.7945	0.7616
	DeeplabV3	0.1177	0.7690	0.8291	0.7721
	PAN	0.1191	0.7573	0.8147	0.7703
	Our	**0.0991**	**0.7888**	**0.8422**	**0.8086**
RGB	Unay et al. [19]	0.2624	0.4546	0.6703	0.4896
	U-Net	0.1683	0.5904	0.6240	0.6838
	U-Net nested	0.1180	0.7330	0.7869	0.7740
	PSP	0.1305	0.7145	0.7794	0.7493
	DeeplabV3	0.1284	0.7452	0.8178	0.7541
	PAN	0.1106	0.7439	0.7717	**0.7919**
	Our	**0.1093**	**0.7938**	**0.8600**	0.7874

**Table 6:** Comparison with hand-crafted and CNN-based methods in the state-of-the-art in terms of RGB + NIR macro class. For each method, *f*-score and *CSRE* are reported. Down-arrow and up-arrow indicates lower is better and higher is better for each metric, respectively. In each column, the best and second-best values are marked in **boldface** and underlined, respectively.

	Defect Class	Unay et al. [19]	U-Net	U-Net Nested	PSP	DeeplabV3	PAN	Our
***f*** **-score ↑**	Bruises	0.6122	0.7978	0.8490	0.8789	**0.9018**	0.8676	0.8729
	Reject	0.6406	0.7271	0.8889	0.8780	0.9271	0.9252	**0.9392**
	Serious defect	0.4632	0.6274	0.7220	0.7179	0.7440	0.7367	**0.7528**
	Slight defect	0.1760	0.4882	0.5525	0.5918	0.5998	0.5929	**0.6660**
***CSRE*** ** ↓**	Bruises	0.2015	0.0836	0.0462	0.0502	0.0358	0.0423	**0.0344**
	Reject	0.1428	0.1366	0.0619	0.0336	**0.0253**	0.0299	0.0321
	Serious defect	0.2539	0.1696	0.1278	0.1253	**0.1123**	0.1174	0.1157
	Slight defect	0.3566	0.2274	0.1612	0.1806	0.1488	0.1625	**0.1211**

**Table 7:** Comparison with hand-crafted and CNN-based methods in the state-of-the-art in terms of RGB macro class. For each method are reported *f*-score and *CSRE*. Down-arrow and up-arrow indicates lower is better and higher is better for each metric, respectively. In each column, the best and second-best values are marked in **boldface** and underlined, respectively.

	Defect Class	Unay et al. [19]	U-Net	U-Net Nested	PSP	DeeplabV3	PAN	Our
***f*** **-score ↑**	Bruises	0.5856	0.7917	0.8849	0.8863	0.8705	0.8912	**0.9051**
	Reject	0.6164	0.6240	0.9032	0.8237	0.9041	0.8596	**0.9404**
	Serious defect	0.5059	0.5362	0.7368	0.7173	0.7114	0.7442	**0.7598**
	Slight defect	0.2197	0.4828	0.5193	0.5285	0.5879	0.5718	**0.6539**
***CSRE*** ** ↓**	Bruises	0.1440	0.1096	0.0475	0.0465	0.0411	0.0557	**0.0321**
	Reject	0.1073	0.2114	0.0516	0.0376	0.0290	0.0665	**0.0266**
	Serious defect	0.1748	0.2170	0.1328	0.1375	0.1248	0.1204	**0.1039**
	Slight defect	0.2574	0.2221	0.1788	0.1960	0.1519	0.1932	**0.1100**

**Table 8:** Comparison with hand-crafted and CNN-based methods in the state-of-the-art in terms of RGB + NIR sub class. For each method, *f*-score and *CSRE* are reported. Down-arrow and up-arrow indicates lower is better and higher is better for each metric, respectively. In each column, the best and second-best values are marked in **boldface** and underlined, respectively.

	Defect Class	Unay et al. [19]	U-Net	U-Net Nested	PSP	DeeplabV3	PAN	Our
***f*** **-score ↑**	Bruise	0.6504	0.7987	0.8938	0.8931	**0.9259**	0.8909	0.9199
	Flesh damage	0.5959	0.8233	0.8717	0.8679	**0.8737**	0.8723	0.8969
	Frost damage	0.5314	0.5978	0.8142	0.8413	0.8382	0.7935	**0.8782**
	Hail damage	0.2176	0.6305	0.6342	0.7169	0.6865	0.7144	**0.7976**
	Hail damage perf	0.5646	0.4971	0.6974	0.6512	0.7594	0.7601	**0.8010**
	Limb rub	0.6660	0.5370	**0.9256**	0.8306	0.8741	0.9016	0.9196
	Other	0.2817	0.4853	0.4338	0.5790	**0.6349**	0.5606	0.5264
	Rot	0.6001	0.7621	0.8551	0.8609	0.8537	**0.8861**	0.8765
	Russet	0.0836	0.5272	0.6400	0.6084	0.6088	**0.6497**	0.6493
	Scald	0.5206	0.5870	0.6996	0.6981	0.7052	0.6137	**0.8016**
***CSRE*** ** ↓**	Bruise	0.1820	0.0857	0.0483	0.0478	0.0362	0.0442	**0.0357**
	Flesh damage	0.1570	0.1169	0.0645	0.0764	0.0693	0.0731	**0.0554**
	Frost damage	0.2417	0.1095	0.0641	**0.0566**	0.0720	0.0860	0.0697
	Hail damage	0.2540	0.1960	0.1178	0.1281	**0.0770**	0.0901	0.0860
	Hail damage perf	0.2451	0.2746	0.1521	0.1265	**0.0879**	0.0906	0.1022
	Limb rub	0.2344	0.2211	**0.0425**	0.1314	0.1073	0.0841	0.0529
	Other	0.3705	0.2390	0.1980	0.1734	**0.1373**	0.1683	0.1403
	Rot	0.1162	0.0987	0.0783	0.0649	**0.0264**	0.0436	0.0645
	Russet	0.3769	0.1413	0.1246	0.1427	0.1647	0.1334	**0.1073**
	Scald	0.2793	0.2608	0.1509	0.1827	0.1244	0.2046	**0.1171**

**Table 9:** Comparison with hand-crafted and CNN-based methods in the state-of-the-art in terms of RGB sub class. For each method, *f*-score and *CSRE* are reported. Down-arrow and up-arrow indicates lower is better and higher is better for each metric, respectively. In each column, the best and second-best values are marked in **boldface** and underlined, respectively.

	Defect Class	Unay et al. [19]	U-Net	U-Net Nested	PSP	DeeplabV3	PAN	Our
***f*** **-score ↑**	Bruise	0.6210	0.7899	0.9048	0.8971	0.9019	0.9066	**0.9170**
	Flesh damage	0.5827	0.7657	0.8736	0.8649	0.8609	0.8494	**0.8779**
	Frost damage	0.5993	0.2797	0.8340	0.7622	0.7302	0.8546	**0.8858**
	Hail damage	0.2999	0.5696	0.6117	0.6308	0.6232	0.6985	**0.7312**
	Hail damage perf	0.5365	0.3664	0.7084	0.6179	0.7824	0.6299	**0.8038**
	Limb rub	0.7914	0.7089	0.9035	0.7964	0.9069	0.9183	**0.9251**
	Other	0.3488	0.5310	0.4374	0.4933	0.5772	**0.6013**	0.5856
	Rot	0.5580	0.7578	0.8771	0.8664	0.8473	0.8716	**0.8793**
	Russet	0.1267	0.4838	0.6547	0.6213	0.6044	0.6122	**0.7076**
	Scald	0.5089	0.6006	0.6430	0.6033	0.7078	0.6488	**0.7370**
***CSRE*** ** ↓**	Bruise	0.1470	0.1145	0.0500	0.0437	0.0430	0.0567	**0.0332**
	Flesh damage	0.1194	0.1578	0.0696	0.0770	0.0724	0.0663	**0.0615**
	Frost damage	0.0928	0.3353	0.0785	0.0456	0.1230	0.0865	**0.0385**
	Hail damage	0.0813	0.2468	0.1045	0.1463	0.1007	0.1288	**0.0821**
	Hail damage perf	0.2136	0.3226	0.1671	**0.0872**	0.0879	0.2048	0.0878
	Limb rub	0.1229	0.2178	0.0579	0.1648	0.0513	**0.0303**	0.0537
	Other	0.2891	0.1680	0.2193	0.2412	0.1502	0.1505	**0.1273**
	Rot	0.0993	0.1347	0.0601	0.0769	**0.0163**	0.0464	0.0490
	Russet	0.2552	0.1762	0.1225	0.1509	0.1513	0.1634	**0.0931**
	Scald	0.2532	0.2408	0.1996	0.2328	0.1430	0.1941	**0.1262**

**Table 10:** The performance of different proposed data synthesis setups. For each algorithm, *CSRE*, *f*-score, *precision* and *recall* metrics are reported. The best configuration (5) is also evaluated in the RGB + NIR scenario. Down-arrow and up-arrow indicates lower is better and higher is better for each metric, respectively. In each column, the best and second-best values are marked in **boldface** and underlined, respectively.

Configuration	Algorithm	Number of Defects	Rotation	Warp	*CSRE* ↓	*f*-Score ↑	*Precision* ↑	*Recall* ↑
RGB	1	-	-	-	0.1110	0.7831	0.8333	0.7856
	2	1	-	-	0.1180	0.7658	0.8405	0.7712
	3	1	✓	-	0.1071	0.7793	0.8423	0.7924
	4	1	✓	✓	0.1269	0.7548	0.8478	0.7526
	5	1 to 3	✓	-	0.1093	**0.7938**	**0.8600**	0.7874
	6	1 to 3	✓	✓	0.1121	0.7812	0.8491	0.7824
RGB + NIR	1	-	-	-	0.1200	0.7680	0.8332	0.7674
	5	1 to 3	✓	-	**0.0991**	**0.7888**	**0.8422**	**0.8086**

**Table 11:** Comparison with hand-crafted and CNN-based methods in the state-of-the-art with the proposed data synthesis procedure. The methods are evaluated in RGB + NIR and RGB configuration. For each method we report *CSRE*, *f*-score, *precision* and *recall*. Down-arrow and up-arrow indicates lower is better and higher is better for each metric, respectively. In each column, the best and second-best values are marked in **boldface** and underlined, respectively.

Image Type	Method Name	*CSRE* ↓	*f*-score ↑	*Precision* ↑	*Recall* ↑
RGB + NIR	U-Net	0.1439 (−7.93%)	0.6040 (−5.51%)	0.6238 (−9.46%)	0.7366 (+4.88% )
	U-Net nested	0.1060 (**−18.27**%)	0.7087 (−2.81%)	0.7067 (−11.07%)	0.8043 (+**7.13**% )
	PSP	0.1229 (−0.89%)	0.7417 (−0.38%)	0.8037 (+1.16%)	0.7630 (+0.18% )
	DeeplabV3	0.1038 (−11.81%)	0.7590 (−1.30%)	0.7872 (−5.05%)	0.8059 (+4.38% )
	PAN	0.1160 (−2.60%)	0.7750 (+2.34%)	**0.8527** (**+4.66**%)	0.7742 (+0.51%)
	Our	**0.0991** (−17.42%)	0.7888 (+**2.71**%)	0.8422 (+1.08%)	**0.8086** (+5.37%)
RGB	U-Net	0.2257 (+34.11%)	0.4552 (−22.90%)	0.5292 (−15.19%)	0.5715 (−16.42%)
	U-Net nested	0.1347 (+14.15%)	0.7375 (+0.61%)	0.8098 (+2.91%)	0.7399 (−4.41% )
	PSP	0.1344 (+2.99%)	0.7440 (**+4.13**%)	0.8224 (+5.52%)	0.7385 (−1.44%)
	DeeplabV3	0.1181 (**−8.02**%)	0.7706 (+3.41%)	0.8535 (+4.37%)	0.7708 (**+2.21**%)
	PAN	0.1248 (+12.84%)	0.7528 (+1.20%)	0.8581 (**+11.20**%)	0.7587 (−4.19% )
	Our	**0.1093** (−1.53%)	**0.7938** (+1.37%)	**0.8600** (+3.20%)	**0.7874** (+0.23% )

**Table 12:** *(Best viewed in colors)*. Methods comparison for CPU and GPU configuration related to RGB + NIR images. FPS = Frame Per Seconds, *CSRE* = Class-Specific Recognition Error, FLOPS(G) = Floating-Point Operations measured in billions (G). Down-arrow and up-arrow indicates lower is better and higher is better for each metric, respectively. For each metric green and red colors indicate best and worst results among all methods respectively.

Method Name	*CSRE* ↓	FPS (CPU) ↑	FPS (GPU) ↑	FLOPS (G) ↓
Unay et al. [19]	0.2523	22.23	27	0.011
U-Net	0.1563	9.2	178	5.769
U-Net nested	0.1297	3.37	74	17.87
PSP	0.1240	5.55	113	14.381
DeeplabV3	0.1177	1.12	22	130.248
PAN	0.1191	2.56	37	47.151
Our	0.0991	8.17	124	7.352

## Data Availability

Data Availability Statement: Data sharing is not applicable to this article.
